# The coexistence of obesogenic behaviors among Brazilian adolescents and their associated factors

**DOI:** 10.1186/s12889-022-13708-6

**Published:** 2022-07-04

**Authors:** Thales Philipe Rodrigues da Silva, Fernanda Penido Matozinhos, Lúcia Helena Almeida Gratão, Luana Lara Rocha, Monique Louise Cassimiro Inácio, Cristiane de Freitas Oliveira, Tatiana Resende Prado Rangel de Oliveira, Larissa Loures Mendes

**Affiliations:** 1 Departamento de Enfermagem Materno Infantil e Saúde Pública, Postdoctoral Fellow, Ph.D in Health Sciences, Child and Adolescent Health, Universidade Federal de Minas Gerais, Escola de Enfermagem, Programa de Pós-Graduação Em Enfermagem, Belo Horizonte, MG Brazil; 2 Maternal and Child Nursing and Public Health Department, Nursing School, Universidade Federal de Minas Gerais, Belo Horizonte, Minas Gerais Brazil; 3 Medical School, Universidade Federal de Minas Gerais, Belo Horizonte, Minas Gerais Brazil; 4 Department of Preventive and Social Medicine, Universidade Federal de Minas Gerais, Belo Horizonte, Minas Gerais Brazil; 5 Nutrition School, Universidade Federal de Ouro Preto, Ouro Preto, Minas Gerais Brazil; 6 Nutrition School, Pontifícia Universidade Católica de Minas Gerais, Belo Horizonte, Minas Gerais Brazil; 7 Nursing Department, Nutrition School, Universidade Federal de Minas Gerais, Belo Horizonte, Minas Gerais Brazil

**Keywords:** Obesogenic behaviors, Obesity, Adolescent

## Abstract

**Background:**

The prevalence of obesity in adolescents has increased significantly in recent years. The growth of obesity is motivated by the association with modifiable behaviors, however, this behavioral are commonly evaluated individually, not considering the possibility of these factors coexisting in the individual. The purpose of this essay was to identify the coexistence of obesogenic behaviors among Brazilian adolescents and to assess the factors associated with the presence of these behaviors.

**Methods:**

This a cross-sectional, national, school-based study with data from the Study of Cardiovascular Risks in Adolescents (ERICA), totaling a sample of 71,552 Brazilian adolescents. To identify the coexistence of obesogenic behaviors in adolescents, the Principal Component Analysis has been performed. To assess the association between factors that influence the coexistence of modifiable behaviors in the pattern of obesogenic behavior, logistic regression was used. The magnitude of the associations was estimated by the Odds Ratio (OR), with the respective 95% confidence intervals (95%CI).

**Results:**

The component was characterized by a higher percentage of ultra-processed food intake, longer in front of screens, having a habit of snacking in front of the television, and not having the habit of eating breakfast. In the adjusted logistic model, it shows that female adolescents and who declare themselves black are more likely to belong to the third tertile of the pattern of obesogenic behavior. As for teenagers who sometimes or almost always or always have lunch or dinner with parents or guardians, who have longer hours of sleep and who live in economically disadvantaged regions have reduced chances of belonging to the third tertile of the pattern of obesogenic behavior.

**Conclusion:**

The identification of obesogenic behavior patterns allows assertive interventions to eliminate or reduce these changeable behaviors, also aiming at the possibility of reducing obesity among adolescents.

## Background

Adolescent obesity rates have been growing all over the world [1, 2], constituting a serious public health issue [3] and one of the greatest global public health challenges of the twenty-first century. The prevalence of obesity in adolescents has significantly increased in recent years, especially in developing countries[1, 4, 5], such as Brazil [5].

Among adolescents, the association of modifiable behaviors with obesity is demonstrated in the scientific literature[6–11]. Among these behaviors, low levels of physical activity and sedentary behavior stand out [12], the consumption of soft drinks and sweetened beverages[13–16], as well as the intake of ultra-processed foods [17, 18]. Changeable behavioral factors are generally assessed individually, not considering the possibility that these factors coexist in the individual.

It is known that reports that consider the coexistence of obesogenic behaviors allow assertive interventions to eliminate or reduce these behaviors, aiming at the possibility of reducing obesity among adolescents [19], since 80% of obese adolescents will remain obese in their age adult [3]. It is noteworthy that the studies that evaluated the coexistence of modifiable obesogenic behavioral factors among adolescents relate their appearance to individual and behavioral characteristics [6–11].

Given the above, this study aimed to identify the coexistence of obesogenic behaviors among Brazilian adolescents and to assess the factors associated with the presence of these behaviors.

## Methods

### Study design, population and data collection

This is a cross-sectional study with data from the Study of Cardiovascular Risks in Adolescents (ERICA). ERICA is a national, school-based, cross-sectional epidemiological study that estimated the prevalence of cardiovascular risk factors and metabolic syndrome in adolescents aged 12 to 17 years who attended public and private schools in Brazilian cities with more than 100,000 inhabitants [20]. The ERICA project, from the Institute for Studies in Collective Health at the Federal University of Rio de Janeiro (UFRJ), is national multicentric research [21].

The researched population was divided into 32 strata, consisting of 27 capitals and 5 sets of counties with more than 100,000 inhabitants in each of the 5 geographic macro-regions of the country. Both sexes, students from public and private schools enrolled in the last three years of elementary school and the three years of high school, morning and afternoon shifts [20].

For each geographic stratum, schools were selected with probability proportional to the size and inversely proportional to the distance from the capital, resulting in a total of 1,251 schools. Schools distributed in 273 Brazilian municipalities were considered, which on July 1, 2009, had more than 100,000 inhabitants, figuring 124 cities. A survey of classes and students of the grades was carried out to allow the selection of three groups of grades per school, with different combinations of time (morning and afternoon) and grade (seventh, eighth, and ninth grade of elementary school and first, second, and third year of High School) [20].

ERICA had 102,327 eligible adolescents, excluding adolescents absent on the day of collection and those who refused to participate in the study. 74,589 adolescents from 1,247 schools in 124 Brazilian municipalities were evaluated. The general collection strategy was coordinated by the ERICA central team, however, in each state, there was a local coordination responsible for all aspects of logistics, for the recruitment and monitoring of supervisors, trained by the central coordination, and for all stages of the process collection of information, which was carried out in schools by contracted and trained field researchers. All students from the selected classes who signed the assent term were interviewed and examined. Adolescents outside the age group of 12 to 17 years who had some degree of disability that made it impossible to perform the anthropometric assessment and fill out the questionnaire, as well as pregnant adolescents [20] were excluded.

In the field collection of ERICA data, three questionnaires were applied: a) adolescents' questionnaire; b) parent/caregiver questionnaire; c) school questionnaire [20]. Adolescents from ERICA answered the self-completed questionnaire on electronic devices (Personal Digital Assistants—PDA) on various topics related to health and lifestyle habits. Data collection took place between February 2013 and November 2014 [20, 21]. For this study, only adolescents who answered the 24-h dietary recall were considered, totaling a sample of 71,552 adolescents.

### Variables’ description

#### Dependent variable

To assess the coexistence of obesogenic behavior, the variables screen hours, snacking in front of the television, breakfast habit, and percentage of ultra-processed food intake were used, which are shown in Table 1. These variables were subsequently used in Principal Component Analysis (PCA) in order to generate one or more patterns of coexistence of obesogenic behaviors.

**Table 1:** Obesogenic behavior indicator variables

Obesogenic behavior	Question in the research	Definition adopted
*Screen hour*	"How many hours do you use the computer, watch TV or play video games on an average weekday?”	Numeric variable
*Habit of snacking in front of the television*	“Do you watch TV eating snacks like popcorn, cookies, snacks, sandwiches, chocolates or candies?”	The answer options were: "I don't watch TV eating snacks", "I watch TV eating snacks sometimes", "I watch TV eating snacks almost every day" and "I watch TV eating snacks every day"
*Habit of eating breakfast*	“Do you have breakfast?”	The answer options were: “I don't have breakfast”, “I have breakfast sometimes”, “I have breakfast almost every day” and “I have breakfast every day”
*Intake of ultra-processed food percentage (UPF)*	Food consumption was assessed using a 24-h recall (24hR) through a face-to-face interview conducted by trained interviewers	Excessive consumption of UPF was considered when consumption was greater than or equal to the 80th percentile of the distribution (45.60% of the total caloric value (TCV)). A large quintile of consumption distribution (P80) was associated with an inadequate food intake profile and a high risk of obesity in previous studies (18)

The variable percentage of ultra-processed food intake is numerical and was obtained through the 24-h recall (24hR). The R24h was applied through interviews conducted by trained researchers [22]. The interview technique used was that of multiple passages, which consists of an interview guided by five steps, intending to reduce underreporting of food consumption [23].

The collected data were registered on small laptops using the Brasil Nutri software. It contained a list of 1,626 foods from the 2002–2003 Household Budget Survey database for food and beverage purchases, carried out by the Brazilian Institute of Geography and Statistics [24]. Foods that were not included in the database were included by the interviewers.

After converting the food items into grams, the data set was linked to the Table of Nutritional Composition of Foods Consumed in Brazil [25] and the Table of Referenced Measures for Foods Consumed in Brazil [26] to obtain the caloric consumption of each teenager. Foods were classified according to the degree of processing, according to the NOVA classification of foods [27]. This classification divides foods into groups according to their nature, extension, and purpose of the industrial processes to which they are submitted. They are fresh and minimally processed foods, processed foods, and ultra-processed foods [27]. Food categorization was performed by two independent researchers. In case of disagreements, an expert researcher was contacted to provide the final result.

For the present study, the percentage variable of intake of ultra-processed foods was generated from the caloric value of all ultra-processed foods ingested by the student and reported in the 24-h recall concerning the total energy intake.

### Independent variables

The independent variables were gender (male and female), self-reported skin color (White, Black, Brown, Yellow, and Indigenous), the habit of having meals with parents (never, sometimes, and always), hours of sleep for the adolescent and the region where the adolescent lives (more economically favored—South, Southeast, and Midwest or less economically favored—North and Northeast, as characterized and used by da Silva et al. [28] and Ricardo et al. [29]).

The variable habit of having meals with the parents was obtained from the questions: “Does your father (or stepfather) or your mother (or stepmother) or guardians have lunch with you”? and “Does your father (or stepfather) or mother (or stepmother) or guardian have dinner with you”?. The answer options were: "my parents or guardian never or rarely have lunch/dinner with me", "my parents or guardian have lunch/dinner with me sometimes", "my parents or guardian have lunch/dinner with me almost every day" and " my parents or guardian have lunch/dinner with me every day”. The answers to the two questions were joined and re-categorized into: "lunch/dinner almost every day or every day" for teenagers who have one of the meals almost every day or every day with their parents or guardian, "lunch/ sometimes have dinner” for teenagers who sometimes have both meals with their parents or guardian, and “never lunch/dinner” for teenagers who never have both meals with their parents or guardian.

The adolescent's hours of sleep variable is numerical and was obtained from the questions: “On a common weekday, what time do you usually sleep”? and “On a typical weekday, what time do you usually wake up”?. To measure the length of sleeping the subtraction between the time the teenager woke up and the time he went to sleep was performed. 24 h were added in situations where negative values were found.

### Variable adjustments

The variable age (12 – 13; 14 – 15; 16 – 17) and wealth proxy were adopted as variable adjustments. The socio-economic classification was defined by ERICA using the Brazilian Economic Classification Criteria (CCEB) of the Brazilian Association of Research Companies (ABEP), in its 2013 version, in which possession of goods (color television, radio) was considered: bathroom, car, refrigerator, freezer, washing machine, and DVD player), presence of a domestic worker, and education of the head of the household. However, in 30.8% of the questionnaires, no information on maternal education was obtained, and the exclusion of these adolescents would imply a significant sample loss.

Therefore, we chose to use the "wealth proxy", as adopted by Moura [30], renamed in this study as socioeconomic score, which was constituted by the CCEB, but considering only the possession of goods and the presence of a domestic worker and has a good equivalence with the ABEP classification. Thus, instead of analyzing the socio-economic classification, the socio-economic score categorized into three equal intervals was used (low socio-economic score: 0 to 12; medium socio-economic score: 13 to 25; and high socio-economic score: 26 to 38).

### Statistical analysis

To identify the coexistence of obesogenic behaviors in adolescents, the PCA was performed. It is an exploratory analytical method that condenses the information contained in the original observed variables into a smaller number of variables, with minimal loss of information. The variables included in the PCA were: hours of screen time, snacking in front of the television, habit of eating breakfast, percentage of ultra-processed food intake, fruit and vegetable intake, and physical activity. However, the variables ingestion of fruits and vegetables and practice of physical activity did not reach satisfactory factor loadings and were removed from the model. The Kaiser-Mayer-Olkin (KMO) coefficient was estimated as a measure of PCA adequacy, with values between 0.5 and 1.0 considered acceptable for this index. Subsequently, components with eigenvalues > 1.0, defined according to the scree plot, were extracted from the PCA.

The component structure was obtained from indicators that presented factor loadings greater than 0.4 or less than -0.4, being a variable generated in scoring scores for the generated obesogenic behavior pattern. After identifying the generated pattern scores, a binary variable was created based on the tertile of the pattern scores, in which the adolescents were categorized as belonging to the 1st and 2nd tertile and as belonging to the 3rd tertile. This binary variable on the pattern of obesogenic behavior was adopted as the dependent variable of the study.

To assess the association between factors that influence the coexistence of modifiable behaviors in the pattern of obesogenic behavior, logistic regression was used. The magnitude of the associations was estimated by the Odds Ratio (OR), with the respective 95% confidence intervals (95%CI). For the multivariate regression model, the backward method was used to build the multivariate model and all variables of interest related to a level of statistical significance below 20% in the bivariate analysis were included in the multivariate analysis, being removed one by one.

Data were analyzed using Stata software, version 16.0. It is noteworthy that, in all analyzes performed, the complexity of the sample was taken into account through the Stata command: svy.

### Ethics approval and consent to participate in the study

ERICA was approved by the Research Ethics Committees of the Institute of Studies in Collective Health of the Federal University of Rio de Janeiro (Report 01/2009), in each state of Brazil and the Federal District. All adolescents who agreed to participate provided written informed consent. Adolescents who agreed to participate in the study have signed the consent form; parents or legal guardians provided written informed consents for all participants younger than 18, according to the ethical guidelines described in Resolution No. 466, of December 12, 2012, of the National Health Council, which involve research with human beings. Participants’ identification remained confidential. All procedures performed in studies involving human participants were in accordance with the ethical standards of the institutional research committee and with the 1964 Helsinki declaration and its later amendments or comparable ethical standards.

## Results

The PCA results are shown in Table 2. From the cutoff point adopted as the scree plot for the Eigenvalue, only the first component was extracted with a total variance of 32.37%. The component was characterized by a higher percentage of ultra-processed food intake, longer in front of screens, having a habit of snacking in front of the television, and not having the habit of eating breakfast. The analysis achieved a satisfactory KMO (above 0.5).

**Table 2:** Factor loadings of the first components of the main component analysis of Brazilian adolescents included in the ERICA study. Brazil, 2013–2014

Indicators	Pattern 1
Percentage of ultra-processed food intake	**0.4062**
Screen hours	**0.5922**
Habit of snacking in front of the television	**0.5686**
Habit of having breakfast	**-0.4013**
Eigenvalue	1.2947
Explained variance (%)	32.37
Overall KMO	0.5569

After identifying the generated pattern scores, a binary variable was created based on the generated tertiles and then the adolescents were categorized into adolescents belonging to the 1st and 2nd tertile (66.67%) and adolescents belonging to the 3rd tertile (33.33%). Most adolescents belonging to the 3rd tertile of the pattern of obesogenic behavior were female (61.32%), of brown skin (51.85%), and aged between 14 and 15 years (39.56%). Regarding the adolescent's daily habits, 63.96% always had meals with the person responsible and slept for an average of 8.62 (SD ± 3.61) hours. Regarding the place of residence, 55.68% lived in an economically favored region and 76.19% were classified as a proxy of average wealth (Table 3).

**Table 3:** Bivariate analysis based on the logistic regression model (OR and *p*-value) of the adolescent's characteristic to pattern 1 (obesogenic behavior) among Brazilian adolescents. – ERICA, Brazil, 2013–2014

Variable	**Obesogenic behavior**^a^	
	n(%)	OR(IC95%)
**Sex**		
Male	8,216(38.68)	Ref
Female	13,025(61.32)	1.42(**1.29 – 1.55**)**
**Skin color (self-reported)**		
White	7,501(36.13)	Ref
Black	1,770(8.52)	1.28(**1.12 – 1.47**)**
Brown	10,766(51.85)	1.09(**1.01 – 1.18**)*
Yellow	563(2.71)	1.04(0.84 – 1.27)
Indigenous	163(0.79)	1.35(0.95 – 1.92)
**Age**		
12 – 13	5,724(26.95)	Ref
14 – 15	8,402(39.56)	1.23(**1.12 – 1.35**)**
16 – 17	7,115(33.50)	1.00(0.90 – 1.12)
**Meals with the guardians**		
Never	2,028(11.22)	Ref
Sometimes	4,484(24.81)	0.82(**0.72 – 0.95**)*
Always	11,558(63.96)	0.66(**0.59 – 0.74**)**
**Sleep time**	8.62(3.61)^b^	0.95(**0.94—0.96**)**
**Favored region**		
Yes (Midwest, South and Southeast)	11,826(55.68)	Ref
No (North and Northeast)	9,415(44.32)	0.64(**0.58 – 0.71**)**
**Wealth proxy**		
High	4,296(21.40)	Ref
Medium	15,297(76.19)	1.07(0.97 – 1.17)
Low	485(2.42)	0.83(0.62 – 1.13)

*Note: OR* Odds Ratio, *95%CI* Confidence Interval

^*^p < 0.05; **p < 0.001

^a^Third tertile of the pattern of obesogenic behavior, *n* = 21.241

^b^Mean (SD)

Bivariate analyzes of the pattern of obesogenic behavior showed an association with female gender, black and brown skin color, age between 14 and 15 years, having meals with parents, longer sleep, and living in a less economically favored region (Table 3).

In Table 4, the adjusted model is described and it was found that female adolescents [OR = 1.51; 95%CI: 1.38–1.66], who declare themselves black [OR = 1.30; 95% CI: 1.12–1.50], aged between 14 and 15 years [OR = 1.17; 95% CI: 1.05–1.30] and which are classified in the mean socio-economic score [OR = 1.20; 95% CI: 1.06–1.36] are more likely to belong to the third tertile of the pattern of obesogenic behavior. As for teenagers who sometimes [OR = 0.82; 95% CI: 0.72–0.93] or almost always or always have lunch or dinner with their parents or guardian [OR = 0.66; 95% CI: 0.58–0.75], who have longer hours of sleep [OR = 0.96; 95% CI: 0.95–0.97] and who live in economically disadvantaged regions [OR = 0.62; 95% CI: 0.56–0.68] have reduced chances of belonging to the third tertile of the pattern of obesogenic behavior.

**Table 4:** Adjusted logistic regression model (OR and *p*-value) of the individual characteristic of the adolescent to obesogenic behaviors among Brazilian adolescents. – ERICA, Brazil, 2013–2014 (*n* = 71,552)

Variable	Obesogenic behavior^a^
	OR_adj_(IC 95%)
**Sex**	
Male	Ref
Female	1.51(1.38 – 1.66)**
**Skin color (self-reported)**	
White	Ref
Black	1.30(1.12 – 1.50)**
Brown	1.06(0.96 – 1.17)
Yellow	1.09(0.82 – 1.45)
Indigenous	1.42(0.96 – 2.10)
**Meals with the guardians**	
Never	Ref
Sometimes	0.82(0.72 – 0.93)*
Always	0.66(0.58 – 0.75)**
**Sleep time**^b^	0.96(0.95—0.97)**
**Favored region**	
Yes (Midwest, South and Southeast)	Ref
No (North and Northeast)	0.62(0.56 – 0.68)**

*ORadj* Adjusted Odds Ratio, 95%CI Confidence Interval

^*^p < 0.05; **p < 0.001

^*^Model adjusted by the presented variables and wealth proxy

^a^Third tertile of the obesogenic behavior pattern

## Discussion

Brazilian adolescents belonging to the third tertile of the pattern of obesogenic behavior consumed more ultra-processed foods, spent more time in front of the screens, had the habit of snacking in front of the television, and did not have the habit of having breakfast regularly. Regarding the factors associated with adolescents belonging to the third tertile of the pattern of obesogenic behavior, in the present study, it was identified that female adolescents who declared themselves black had increased chances of belonging to the pattern of obesogenic behavior. On the other hand, those adolescents who ate meals with their parents or guardians sometimes, almost always or every day, who had longer sleep duration and who lived in less economically favored regions (North and Northeast) showed a reduction in the chances of belonging to a pattern of obesogenic behavior.

In the present study, the variables screen hours, snacking in front of the television, breakfast consumption, and percentage of ultra-processed food intake were evaluated to verify the existence of obesogenic behavior. The coexistence of obesogenic behaviors in adolescence, as well as their association with sociodemographic characteristics, behaviors, and health outcomes, has been the subject of studies in recent years [6–9, 11, 19, 31], not being a reality only for Brazilian adolescents [32, 33]. In this context, the importance of studying this topic for the prevention and treatment of obesity and other chronic non-communicable diseases (NCDs) is highlighted. However, the comparison of the results of this study with others should be interpreted with caution, due to the different methods of statistical techniques used to identify the coexistence of obesogenic behavior [6].

It is known that obesity prevention initiatives conducted in an isolated way at adolescents do not achieve effective weight loss [34, 35]. Identifying the coexistence of obesogenic behaviors in adolescence allows for interventions in multiple modifiable behaviors and, consequently, increases the probability of reducing obesity rates among adolescents [36].

Regarding sociodemographic characteristics, the coexistence of obesogenic behavior was associated with the female gender. This result is consistent with previous studies that found a greater presence of risk factors for the development of NCDs and obesity among girls [6, 37, 38]. Adolescents, in general, tend to adopt unhealthy behavior patterns [39], especially girls. At this stage, the adolescent undergoes intense physical, psychological, social, dietary, and lifestyle changes [40, 41]. Among female adolescents, studies show that they tend to have less healthy eating habits [32, 42], characterized by high consumption of ultra-processed foods and low consumption of healthy foods [42, 43].

Regarding the racial disparities found in this study, black-skinned adolescents were more likely to have coexistence of obesogenic behaviors. Studies [9, 44–46] demonstrate that ethnic and racial minorities experience a high prevalence of obesogenic behavior and obesity. Racial disparity is something to be overcome since they persist even after adjusting the adolescents' socioeconomic status indicators [46].

Adolescents who lived in economically disadvantaged regions (North and Northeast) showed a reduction in the chances of belonging to a pattern of obesogenic behavior. Previous studies on risk factors for NCDs and cardiovascular diseases show similar findings, in which the coexistence of three or more risk factors was more frequent among adolescents living in cities in more developed urban areas of the country [28, 29]. Data from the Household Budget Survey (HBS) carried out in Brazil between 2017 and 2018 showed there was an increase in the consumption of AUP in the more socioeconomically developed regions of Brazil [47]. However, this finding is contrary to the previous study with Brazilian adolescents from the National Survey of School Health (PeNSE) in 2009, where the association was inverse [6] to that found in this study.

Concerning the habits of adolescents, those who sometimes or almost always or always eat with their parents or guardian showed a reduction in the chance of having coexistence of obesogenic behaviors. Studies show that the habit of having meals with the guardians is a protective factor against obesity [48–50]. In a systematic review carried out by Amaral et al. [50], it was found that eating meals with parents or guardians, favors the adoption of healthier eating patterns, with increased consumption of fruits, vegetables, whole grains, and beans. Having meals with parents increases parental control over food intake [51] of adolescents. In this sense, parental support and control are important for the formation of eating habits of adolescents, in addition to the adolescents' own awareness of obesogenic behavior [6].

Finally, sleep duration influenced the reduction in the chance of adolescents having coexistence of obesogenic behaviors. Previous studies have already linked sleep duration with the onset of obesity [52–55] among adolescents [54]. It is known that sleep duration influences an adolescent's eating pattern. Short sleep duration plays an important role in the onset and development of obesity through changes in endocrine, neurological and behavioral mechanisms [55]. In addition, sleeping less increases the probability of individuals to snack and consume foods with high energy density [55]. A study carried out in Spain shows that sleeping for a sufficient number of hours (greater than 9.9 h/day) was associated with higher consumption of fruits and vegetables [56].

This study has some limitations, such as the “social desirability” bias, that is, the possibility that adolescents tend to respond, in the questionnaire, to previously standardized and well-accepted social behaviors. However, the adolescents were informed about the anonymity of the responses. The second limitation refers to the fact that behaviors are self-reported, which may have led to an information bias, possibly underestimating the prevalence of risk behaviors. Furthermore, there is a limitation by the use of a 24-h Reminder, which may not represent the usual intake. However, the potential of the study is given by the sample representativeness, for having external validity and allowing generalization to the Brazilian population of adolescents between 12 and 17 years of age in Brazilian cities with more than 100,000 inhabitants, as the ERICA study is school-based with national representation for the population.

This study advances in identifying the coexistence of obesogenic behaviors in Brazil and is the first work to be carried out using ERICA data to identify this pattern. Furthermore, it advances in identifying the influence of sociodemographic variables and individual behaviors of Brazilian adolescents on the coexistence of risk factors.

## Conclusions

Given the above, this study highlights the coexistence of obesogenic behavior observed in Brazilian adolescents whose behavior patterns include a greater intake of ultra-processed foods, longer time in front of screens, the presence of snacking in front of the television, and not eating breakfast regularly. From the logistic regression analysis, it is evident that being female and with black skin color have increased chances of having a pattern of obesogenic behavior. Having meals with parents or guardians, longer sleep duration, and living in less economically favored regions of Brazil (North and Northeast) presented a reduction in the chances of having a pattern of obesogenic behavior.

## Data Availability

The datasets used and/or analysed during the current study will be available from the corresponding author on reasonable request.
